# Image based evaluation of mediastinal constraints for the development of a pulsatile total artificial heart

**DOI:** 10.1186/1475-925X-12-81

**Published:** 2013-08-14

**Authors:** Andreas Johann Fritschi, Marco Laumen, Sotirios Spiliopoulos, Thomas Finocchiaro, Christina Egger, Thomas Schmitz-Rode, Gero Tenderich, Reiner Koerfer, Ulrich Steinseifer

**Affiliations:** 1 Department of Cardiovascular Engineering, Institute of Applied Medical Engineering, Helmholtz Institute, RWTH-Aachen University, Aachen, Germany; 2 Department for the Surgical Therapy of end-stage Heart Failure and Mechanical Circulatory Support, Heart- and Vascular Center Duisburg, Duisburg, Germany

**Keywords:** Artificial heart, Prosthesis design, Image reconstruction, Three-dimensional, Computer-aided design, Biomedical engineering

## Abstract

**Background:**

Good anatomical compatibility is an important aspect in the development of cardiovascular implants. This work analyzes the interaction of the pump unit of an electrically driven pulsatile Total Artificial Heart (TAH) and the mediastinum. For an adequate compliance, both overall dimensions and alignment of inlets and outlets must be matched.

**Methods:**

Cross-sectional medical image data of 27 individuals, including male and female patients suffering from end stage heart failure, was segmented and reconstructed to three dimensional (3D) surface models. Dimensions and orientations of relevant structures were identified and analyzed. The TAH surface model was virtually placed in orthotopic position and aligned with atrioventricular valves and big vessels. Additionally seven conventional cadaver studies were performed to validate different pump chamber designs based on virtual findings. Thereby 3D-coordinates were captured and introduced to the virtual environment to allow quantitative comparison between different individuals.

**Results:**

Spatial parameters varied more in male patients with higher values if heart failure persists. Good correlation of the virtual analysis both to literature data and conventional cadaver studies could be shown. The full data of the 27 individuals as well as the summarized values found in literature are enclosed in the appendix. By superimposing the TAH-volume model to the anatomy, various misalignments were found and the TAH-design was adjusted.

**Conclusions:**

Virtual fitting allows implant design adjustments in realistic anatomy which has not been influenced by thoracotomy. Higher numbers of relevant individuals can be reasonably investigated in the virtual environment and quantitatively correlated. Using this approach, conventional cadaver studies can be significantly reduced but not obviated, due to the unavailable haptic feedback and immobility of potentially compressed structures.

## Background

A Total Artificial Heart (TAH) replaces the human heart both functionally and anatomically. In some cases a TAH can be an alternative to orthotropic heart transplantation for terminal heart failure patients [1]. When intended to be used for destination therapy, there are advantages if the system is designed to be fully implantable. Without a skin penetrating driveline, patient mobility is increased and critical infection spots are avoided. The drawback of such a setup is that the successfully proven pneumatic drive concept [2] cannot be used anymore. Therefore, not only the chambers, but also the drive unit and further components dealing with energy transmission, have to be placed intracorporeal. It has been shown that it is possible to transmit sufficient electrical energy transcutaneously using two induction coils [3], which is then converted to a mechanical movement by the drive unit. In pulsatile TAHs this drive unit is typically placed in between or in close proximity to the systemic and pulmonary pump chambers. However, one severe limitation of a TAH system is the implant size of the thoracic unit itself. This work focuses on a virtual fitting study for the pump unit of the Aachen TAH ReinHeart, which comprises a direct linear drive [4,5], and examines the available space within the human thoracic cavity and the connector configuration of the in and outgoing blood vessels in a relevant patient population. The ReinHeart TAH system is in development stage and currently undergoing short term chronic *in-vivo* studies.

The study was conducted using the following course of action: The virtual device placement determined the initial design of the implant in a small group (n = 2) of individuals, which was subsequently verified in four conventional cadaver studies. Since there were variations within these studies, a higher number of individuals (n = 25) was virtually investigated and compared to literature. A comprehensive list of both the achieved data as well as the reviewed and averaged literature can be found in the attachment. To coincide with the resulting outcome, small adjustments to the implant design were made and again verified in three cadaver studies. In these studies 3-dimensional coordinates of the present structures were captured. These findings were introduced to the virtual environment and correlated to the virtual fitting data.

This is the first depiction, where quantitative capturing of coordinates during a cadaver study was realized, which allowed the creation of a virtual model and therefore a direct, quantitative comparison of radiological images and cadaver results.

## Methods

### Virtual analysis

High resolution sectional image data acquired by computer tomography (CT) scans of 27 patients (15 female, 12 male) were retrospectively and anonymously investigated. To achieve good representations of relevant structures, all scans were acquired in end diastolic phase during apnea of the patient. Sixteen of these patients had some degree of Aortic Valve (AV) stenosis (Group: av), six had an unknown medical history (Group: no-hx), and five were potential candidates for a mechanical circulatory support device (Group: mcs - for details see Additional file 1: Appendix A).

**Figure 1:**
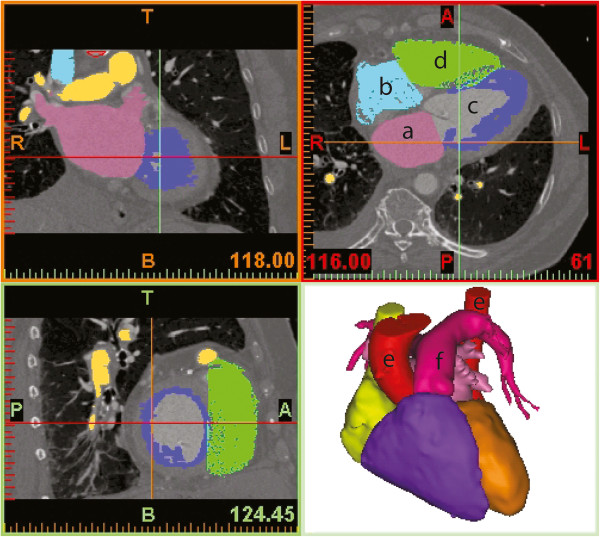
**Segmenting software (Mimics 13.1) with six masks of structures of interests segmented and reconstructed.** Upper left: frontal view; Upper right: transversal view; Lower left: sagital view; Lower right: 3D reconstruction. Visible structures of interest: left atrium **(a)**, right atrium **(b)**, left ventricle **(c)**, right ventricle **(d)**, aorta ascendens **(e**1**)** and descendens **(e**2**)** (arch outside field of view), pulmonary artery **(f)**.

The existent data was transmitted to a commercial software package (Materialise Interactive Medical Image Control System (Mimics) 13.1, Materialise NV, Leuven, Belgium) using the Digital Imaging and Communications in Medicine (DICOM) standard. The structures of interest were the left and right atrium, left and right ventricle, pulmonary artery, aorta, myocardium, lungs, diaphragm, and thoracic cage (sternum, thoracic vertebrae, and ribs). To isolate and segment these structures, a Hounsfield unit threshold mask was applied, followed by automated standard image processing tools such as static and dynamic region growing, and morphology operations as well as manual editing of the slices. To increase reproducibility, it was avoided to manually modify single pixels but rather use an existent algorithm whenever possible. The final mask of each structure was then reconstructed into a three dimensional surface data set, visualized as a surface rendering (see Figure 1 lower right), tessellated, and saved in standard triangulation format. Depending on the quality of the scan, the segmentation process required about eight hours of time for one patient, assuming the user is experienced. This data was then transmitted to 3matic 5.0 (Materialise NV, Leuven, Belgium), which enables standard Computer Aided Design (CAD) operations on freeform shape geometries such as anatomical surface models. A surface smoothing process consisting of three iterations of a first order Laplacian smoothing algorithm (factor 0.7; compensate shrinkage enabled) followed by the wrapping tool to close minimal gaps smaller than 0.5 mm (protect thin walls enabled), and three iterations of the quality preserving reduce triangles tool with a tolerable maximum geometrical error of 0.1 mm (shape quality threshold: 0.3) was applied to eliminate small irrelevant inconstancies due to image noise. Due to the complexity of the models and the therefore necessary computational time in between single steps, this process required about four hours of time per patient. Thereafter these models were analyzed, which included taking both oblique two-dimensional measurements such as distances and diameters as well as angles and directions; in the latter case normal vectors were recorded.

The first set of parameters investigated the spatial constraints, which correspond to the pericardial cavity, and determines the overall dimensions of the implant. Since it is not feasible to identify the pericardial sac itself on radiological images, a three dimensional model of the encircling structures such as the sternum, the vertebrae, the pulmonary lobes, the diaphragm, and the great vessels was realized and the space in between was investigated. In literature the cranial-caudal distance is described by two different approaches. If identified on radiological images [6-9], the measurement was taken from aortic valve to diaphragm. If taken intraoperatively after resection of the ventricles during heart transplantation [10,11], the value from the remnant aorta (distal to aortic valve, proximal to brachiocephalic artery) to the diaphragm was considered. To allow a later comparison both values were noted. The lateral dimension was taken from the right edge pericardium to the left edge pericardium at its widest point. The ventral-dorsal dimension was also measured and recorded using two different definitions: The first definition describes the distance from the dorsal side of the sternum to the posterior pericardium, taken at its widest point in space. The second definition describes the distance between the 10th thoracic vertebrae (Th10) and the sternum in the transversal plane and was recorded due to its more practical use to ease application for other groups, although it probably is less precise than the before mentioned.

**Figure 2:**
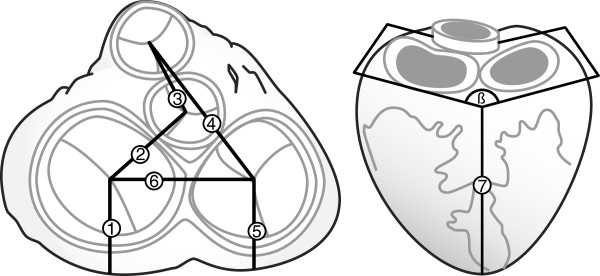
**Investigated valve parameters (based on figure three in **[10]**).**

The second set of parameters is related to valve diameters, distances, and orientations. Therefore, an inscribed circle was fitted in the most proximal region of the leaflets geometry in an oblique manner. Diameters of the Mitral Valve (MV), Tricuspid Valve (TV), Pulmonary Valve (PV), and Aortic Valve (AV) as well as the distances in between valve centers (MV to AV, AV to PV, PV to TV, and TV to MV - see Figure 2 no. 2,3,4, and 6) were taken. To investigate how the valves are positioned in the pericardial space, the distances between the four valves and the apex as well as the longitudinal distention (atrial septum to apex) (see Figure 2 no. 7) was considered. Since the atrioventricular (inlet) valves are embedded within their surroundings, the angle in between the atrial orifices (see Figure 2 ß) and the distance from the posterior surface of the pericardium to the center of TV and MV (see Figure 2 no. 1 and 5) was noted as well. In contrast the position of the semilunar (outlet) valves can be adjusted to some degree by mobilizing the efferent vessels, which makes a reliable and reproducible measurement impractical and was therefore discarded.

### Virtual fitting

**Figure 3:**
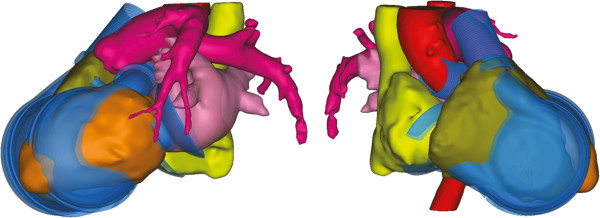
Left and right chamber with adapted in and outlet configurations superimposed to anatomical structures of female patient number 14 (mcs group).

The initial design of the pump chambers was exported from the Computer Aided Design (CAD) program Pro Engineer Wildfire 3.0 (Parametric Technology Corporation, Needham, MA, USA) in standard triangulation format, and superimposed on the physiological structures in 3matic by translating and rotating the pump unit into the orthotopic position. The now apparent misalignments of the chamber design were corrected in the CAD software in several iterations until a good comprehensive agreement (see Figure 3) for the first two datasets was found.

### Cadaver fit evaluation

During the design process of the pump chambers a total of seven studies were performed using freshly preserved adult corpses. Subsequently to median sternotomy and incision of the pericardium, the native right and left ventricles were excised at the atrioventricular plane whereby special care was taken that the annulus of the mitral and the tricuspid valves along with a small rim of ventricle muscle were preserved. Based on earlier described approaches [12], the atrial cuffs were sutured and the outflow grafts anastomosed to the aorta and the pulmonary artery.

**Figure 4:**
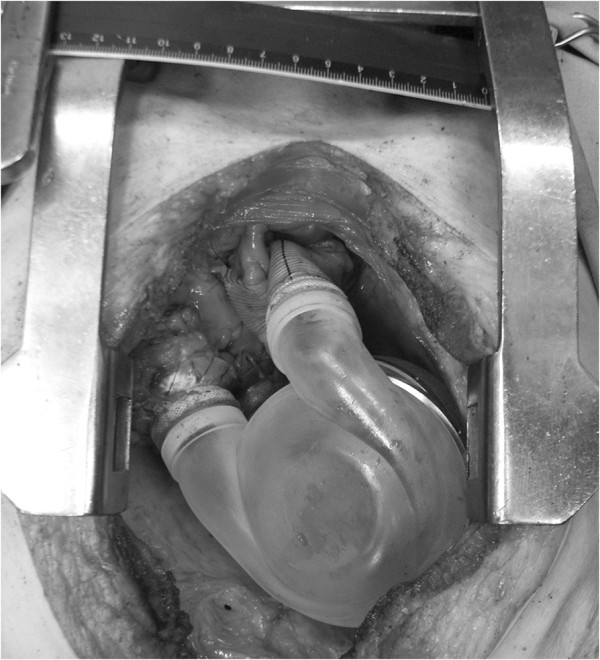
**Anastomosed cuffs and grafts with manufactured volume model *in-situ *(cadaver study 2).**

Using additive manufacturing methods, better known as rapid prototyping, (FullCure720 material, Eden350V; Objet Geometries Ltd., Rehovit, Israel) it was possible to realize new and improved models with slightly modified positions and orientations of the in- and outlet of the chambers between each cadaver study (see Figure 4).

Additionally a new version of the drive unit with a reduced outer diameter of 82 mm (originally 90 mm) became available [13] and could immediately be integrated. Whereas the arrangement of the ducts within one chamber was fixed during the actual trial, there still was one degree of freedom between the two chambers, which is the rotation around the centerline. After accurate adjustment the current angular offset of both chambers could be read off from an imprinted scale to allow for later comparison. The main intention of the first four cadaver studies was to see how well the implant model interacts with the mediastinum and to gain some haptical feedback. Some very basic distance measurements were manually read out using scale paper.

### Cadaver analysis

**Figure 5:**
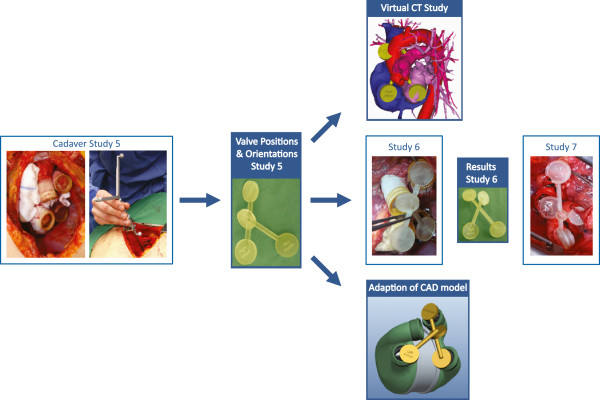
**Quantification of cadaver study five (left), manufactured valve model (middle) and comparison with: 1) Reconstructed CT scans of different individuals (right above), 2) Subsequent cadaver studies six and seven (right middle), 3) Current technical implant design (CAD model- see right below) (modified from **[14]**).**

Since the acquired values were not very accurate, an optical 3D- coordinate measuring system (Polaris Vicra, Northern Digital Inc., Waterloo, Canada) was used to capture the position and direction of the prostheses in cadaver study five, six and seven (two male, one female – for age and BSA see results section). Therefore, manufactured cylinders with a defined center and four encircling slots were inserted into the anastomosed grafts and cuffs. The pointer device of the Polaris system, which is a known rigid geometry with three reflecting spheres, was used to get the absolute coordinates of these points (see Figure 5 left). Using vector algebra, the normal vectors were calculated, and computational geometries were generated (3matics 5.1, Materialise NV, Leuven, Belgium).

The resulting valve model was compared to both reconstructions of other individuals CT data (see Figure 5 right above) as well as the current technical CAD geometries (see Figure 5 right below) Additionally the digital model was manufactured (FullCure720, Eden350V) and used for comparison in subsequent cadaver studies (see Figure 5 right middle). According to the guidelines of the responsible Ethics Committee approval is not required for anonymized and retrospectively used images. The cadaver study was performed in cooperation with the Institute of Molecular and Cellular Anatomy of the RWTH Aachen University.

## Results and discussion

**Figure 6:**
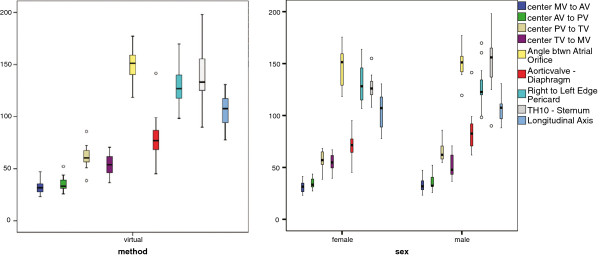
**Box plot of crucial parameters of virtual analysis.** Left: summarized results of virtual study (n = 27). Right: results of virtual study dependent on gender (n = 15 female; 12 male).

**Figure 7:**
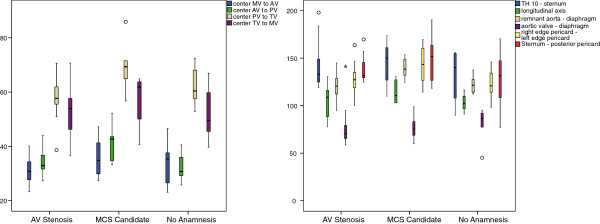
**Analyses according to the three patient groups Aortic Valve Stenosis, potential candidates and unknown medical history.** Left: distances between valve centers. Right: spatial parameters.

The different parameters of the virtual analysis are summarized, and distributions of the relevant factors are depicted in box plots (see Figures 6 and 7 - SPSS 18.0, International Business Machines Corp, Armonk, NY, USA). The results are compared to literature and, where possible, to cadaver parameters. Furthermore, the development and the final findings for the left and right pump chambers are described and discussed.

### Virtual analysis and analogy to literature

The examined parameters varied from 11% and 32% between different individuals, whereat variations were higher in male than female patients (p = 0,032). Relevant valve parameters such as distances between valve centers and the inlet orifice angle are relatively uniform between both sexes, whereas several spatial parameters differ. Thereby the cranial-caudal as well as the ventral-dorsal extension was about 10% higher in male than female patients, whereas the lateral extension was congruent.

The values found in literature were averaged from a total of up to 67 individual patients with different backgrounds, and were acquired using different modalities and techniques (for details see Additional file 2: Appendix B). Shah (n = 12) and Shiono (n = 26) took parameters intraoperatively during an orthotopic heart transplantation (HTX) surgery [10,11], Chatel (n = 15 ) used CT images of HTX candidates [9], and Komoda in his three studies (n = 3;6;5) used data acquired by Magnetic Resonance Imaging (MRI) of healthy individuals [6-8]. For the cranial-caudal parameter (see “+” in Table 1) only the values from Komoda were considered, since the others were either not available or not appropriate (remnant aorta during HTX). The Th10-sternum parameter was not available in all cases; therefore, the anterior-posterior value of the myocardium was quoted (see “*” in Table 1).

**Table 1:** List of crucial parameters: Results of virtual study and comparison to literature

**Parameter**	**Virtual**	**Virtual**	**Virtual**	**Literature**
	**Total**	**Male**	**Female**	**Total**
Center MV to AV	32	33	32	40
Center AV to PV	35	36	35	32
Center PV to TV	61	64	58	66
Center TV to MV	53	51	55	52
Angle atrial orifices	149	150	148	161
AV-diaphragm	77	85	70	78+
Right to left pericardium	129	128	130	126
TH10 - sternum	141	155	130	111*
Longitudinal axis	106	108	106	107

“+” only values from the three Komoda studies are considered.

“*” the anterior-posterior value of the myocardium is quoted instead of the TH1—sternum.

The acquired values of most parameters are in good agreement with literature with a deviation of less than 10%. These are the cranial-caudal and the lateral distance, the orifice angle, and three out of the four valve distances. Exceptions are the distance between mitral and aortic valve with a deviation of 20%, and the Th10 to sternum distance, which differs by 26%. One possible reason for the former misalignment may be the different interpretation of the center point of the noncircular, but elongated atrioventricular valves by different groups (e.g. inscribed circle, gravity center). More arguments are the change in distance due to movement of the mitral valve, which was already ascertained to be up to 18 mm by one of the groups [7], or the more distal measurement on excised hearts during HTX. However, since all this is the case for the TV as well, this requires further investigation. When analyzing the latter parameter, it is clear, that Shiono, Shah, and Chatel have similar values, which average 133 mm contraire to Komoda, who reported 88 mm. Our consideration is that this may be due to a combination of patient anamnesis, age, and ethnic background. In both cases the width of the descending aorta has to be added to be comparable to the Th10-sternum value.

When considering the distinct patient groups (see Figure 7), the variations within the AV group as well as the no-hx group are identical to the overall results. Solely the values of the mcs-candidates deviated somewhat less, despite the fact that the age of these patients varied most and ranged from 45 to 98.

When comparing single parameter among the different groups and averaging the deviations, the values of the mcs group are 8% higher compared to the no-hx group and the averaged literature and even 11% higher than the values of the av group. This is the case for both sets of parameters, but is most evident in the distance between the apex and the four centers of the valves. One notable exemption here is the consistent atrial orifice angle beta along with the distance in between mitral and tricuspid valve, whereas the distance between every other valve is increased. It could also be observed that the diseased myocardium does not dilate uniformly, but most rapidly in cranial-caudal direction. This dimension increased by 17% compared to a 12% lateral and 9% ventral/dorsal increase.

### Analogy between virtual and cadaver studies

As mentioned above, during the cadaver studies the connector of the attached prostheses was detected instead of the physiological valve. Therefore, a direct comparison is inappropriate. The divergence within these values was higher than *in-silico* (see Table 2). One reason may be the varying anastomosis of the prostheses to the tissue by different surgeons. Another reason could be the high mobility of the attached tubular graft prostheses, as in these recordings the values differ most. However, due to the small number of cases (n = 3) and the gradual protocol development, these findings should be considered with care.

**Table 2:** Results cadaver study

**Sex / patient_ID**	**Cmale5**	**Cfemale6**	**Cmale7**
**Anamnesis**	**Unknown**	**Unknown**	**NYHA3**
**Age**	**65**	**70**	**81**
**BSA**	**1,9**	**1,4**	**2,0**
MV to AV	77	59	53
AV to PV	25	33	49
TV to PV	60	72	50
MV to TV	47	50	28
Angle atrial orifices	158	159	135
AV-diaphragm	NA	69	103
Right to left pericard	NA	124	138
TH10 - sternum	NA	98*	142*
Longitudinal axis	NA	NA	106

“*” the anterior-posterior value of the myocardium is quoted instead of the TH1—sternum.

To compare both virtual and cadaveric measurements of one individual, cadaveric CT-scans were inspected. Due to the absence of contrast agent, they were found to be not helpful in these primarily vascular considerations.

### Pump chamber evolution

**Figure 8:**
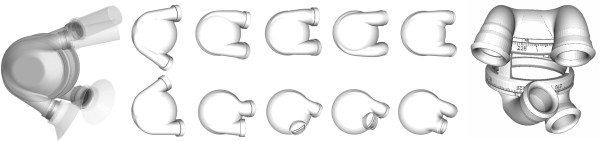
**Pump chamber designs.** Left: CAD model (lumen) of initial left and right pump chamber with attached cuffs and grafts. Middle: important intermediate steps of the iterative evolution of right (upper row) and left (lower row) pump chamber design, these steps were manufactured and used in the cadaver studies. Right: resulting revolvable volume model with imprinted angle scale.

Anatomical considerations conducted prior to this study limited the overall dimensions of the TAH pump unit to a maximum diameter of 84 mm and a height of 90 mm. The in and outlet valves were appointed to be contorted between 45 to 90 degrees [15]. To achieve good hemocompatibility, this initial geometry was then optimized to avoid stagnation areas as well as potential flow disturbances. Therefore, a tangential junction of both the inlet and the outlet duct to the rotation symmetric base body was applied (see Figure 8 left). It has been shown that for this geometry one extensive central vortex is generated, which provides good wash out performance [16]. Despite the inversely placed valves, the left and right chambers were similar in the initial concept.

When analyzing this concept in both virtual as well as following cadaver studies, it was found that the distance in between the inlet valves should be reduced.

Therefore, the right inlet was shifted as close as possible towards the drive unit without causing an obstruction. Since this was not sufficient due to the sandwiched drive unit design [4], it was additionally tilted towards the midplane. For an improved coincide with the right myocardium, the aperture angle of the right outlet duct was reduced while maintaining the tangential junction. To compensate for the newly formed misalignment with the pulmonary artery, the distal end of the outlet duct was curved away from the center line. The iterative evolution of the right chamber can be traced in the upper row of Figure 8.

By superimposing and aligning the initial left pump chamber design to the reconstructed image data, it became evident that the distance between the two ducts was too large. When repositioning the left inlet valve closer towards the outlet valve by maintaining a tangential duct, virtual pinching of the left atria occurred. Due to the higher physiological demand, a decrease in filling and therefore insufficient cardiac output is especially critical in the left chamber. Neglecting optimal washout performance, the inlet valve was therefore placed directly at the base body of the left chamber, which allowed for a contraction in length. To align with the left atrium (see Figure 8 right), it was shifted 10 mm lateral and tilted about 25 degree towards the middle axis. To match the ascending aorta, the outlet duct had to be inflected towards the center plane. In situ, the aortic graft can then be placed caudal to the pulmonary graft, which represents the physiologic situation (see Figure 3 left). The iterative evolution of the left chamber can be traced in the lower row of Figure 8.

Using the final findings of both redesigned chambers, a revolvable model was created (see Figure 8 right) and manufactured, which represents the whole thoracic pump unit including the drive unit, the two pump chambers and the electric and pneumatic connectors. Using the imprinted scale a defined rotation of both chambers can be set and validated *in-situ*. In the future this model may also be used as an implantation dummy in *in-vivo* studies to enhance the placement of the prostheses during the operation.

## Conclusion

The achieved results from virtual and cadaver studies are congruent to the investigated literature. The in- and outlet configurations of the optimized TAH design could be closely matched to the physiological parameters. To adapt for minor variations occurring in different individuals, a range of vascular prostheses (grafts and cuffs) can be used.

By using the above described approach, investigation of least invasive placement and therefore necessary design adaptations of additional intracorporeal components of the TAH-system are currently in progress. In particular these are the primary TET-coil, the drive unit controller, the internal buffer battery, and the volume displacement chamber, which is necessary to avoid pulmonary decompensation.

Virtual Fitting of cardiovascular implants based on medical imaging data is a powerful tool in the development of new devices. This method can not only be used for preliminary design optimization of medical devices, and thus reducing the number of cadaver studies, but also provides a method to examine fitting in anatomical environment unaltered by sternotomy. The correlation to conventional fitting studies could be successfully shown. One severe drawback in comparison to conventional studies is the unavailable haptic feedback of necessarily compressed structures and therefore the estimation of the resulting impact due to this alteration.

Transferring this technique to a clinical setting would also be conceivable by using CT scans of each individual patient. The conducting heart surgeon may then be assisted in planning the placement of the system components before the implantation procedure is initiated. A similar approach has been investigated by Dowling et al for implanting the Abiocor TAH [17-19]. These authors reveal virtual implantation planning as, “a powerful tool that we have become reliant on for selection of patients“ from [19]. Unfortunately, the procedure itself is described only ambiguously in reviewed literature. The most elaborate allusions are given in the Abiocor Instructions for Use (accessible through FDA website), however this information is not sufficient to allow a reproduction by others. Therefore the detailed description of the methodology within this work, which also mentions the used parameters of the performed steps, is considered to be of benefit for other groups and may ease correlation of similar findings in the future.

If image processing is further simplified, anatomical contraindications for a TAH implantation could be made visible preoperatively onsite in a clinical setting. By superimposing digital volume models of various pump devices this approach could even help to choose the appropriate system with respect to each individual’s anatomy.

## Abbreviations

3D: Three dimensional; AV: Aortiv valve; av: AV stenosis group; BSA: Body surface area; CAD: Computer aided design; CT: Computer tomography; DICOM: Digital imaging and communications in medicine; FDA: Food and drug administration; HTX: Orthotopic heart transplantation; mcs: Mechanical circulatory support group; Mimics: Materialise interactive medical image control system; MRI: Magnetic resonance imaging; MV: Mitral valve; no-hx: Unknown medical history group; PV: Pulmonary valve; SPSS: Statistical product and service solutions; TAH: Total artificial heart; TET: Transcutaneous energy transmassion; Th10: 10th thoracic vertebrae; TV: Tricuspid valve.

## Competing interests

The authors declare that the research was conducted in the absence of any commercial or financial relationships that could be construed as a potential conflict of interest.

## Authors’ contributions

Each Author has contributed substantially to the research, preparation and production of the paper and approves of its submission to the Journal. All authors read and approved the final manuscript.

## Supplementary Material

Additional file 1: Appendix A Results of virtual study in detail. Click here for file

Additional file 2: Appendix B Summary of data found in literature. Click here for file
