# Supplementary material for: Image based evaluation of mediastinal constraints for the development of a pulsatile total artificial heart
Source: Biomed Eng Online. 2013 Aug 14;12:81. doi: 10.1186/1475-925X-12-81 (PMC3751639; doi:10.1186/1475-925X-12-81)
Supplement: Additional file 1: Appendix A — Results of virtual study in detail. [file 1475-925X-12-81-S1.docx]

Additional file 1: Results of virtual study in detail

Appendix A part 1: female patients

| **sex / patient_ID** | **fem1** | **fem2** | **fem3** | **fem4** | **fem5** | **fem6** | **fem7** | **fem8** | **fem9** | **fem10** | **fem11** | **fem12** | **fem13** | **fem14** | **fem15** | **Avg.Female** |
| --- | --- | --- | --- | --- | --- | --- | --- | --- | --- | --- | --- | --- | --- | --- | --- | --- |
| **group** | **av** | **av** | **av** | **av** | **av** | **av** | **av** | **av** | **av** | **av** | **av** | **no hx** | **no hx** | **mcs** | **mcs** | **n=15** |
| **age** | **82** | **79** | **80** | **88** | **91** | **82** | **85** | **90** | **78** | **88** | **87** | **69** | **85** | **55** | **98** | **82** |
| **scan resolution** | **0,43** | **0,49** | **0,49** | **0,45** | **0,39** | **0,51** | **0,4** | **0,57** | **0,45** | **0,36** | **0,39** | **0,4** | **0,7** | **0,39** | **0,46** | **0,46** |
| **slice Increment** | **0,3** | **0,3** | **0,3** | **0,3** | **0,3** | **0,3** | **0,3** | **0,3** | **0,3** | **0,3** | **0,3** | **0,33** | **0,63** | **2,5** | **1** | **0,52** |
| aortic valve-diaphragm | 55 | 64 | 88 | 95 | 73 | 68 | 59 | 71 | 61 | 67 | 77 | 77 | 45 | 83 | 72 | 70 |
| remnant aorta-diaphragm | 96 | 113 | 145 | 120 | 120 | 125 | 123 | 144 | 106 | 111 | 126 | 125 | 114 | 125 | 139 | 122 |
| right to left edge pericard | 123 | 137 | 164 | 129 | 127 | 132 | 147 | 127 | 107 | 115 | 110 | 114 | 146 | 146 | 127 | 130 |
| ventral-dorsal | N/A | 137 | 125 | 127 | 132 | 129 | 130 | 131 | 128 | 132 | 146 | 109 | 147 | 118 | 127 | 130 |
| TH10 – sternum | 158 | 139 | 119 | 125 | 130 | 139 | 124 | 120 | 133 | 131 | 125 | 108 | 155 | 110 | 127 | 130 |
| diameter MV | 34 | 26 | 29 | 24 | 31 | 29 | 31 | 30 | 25 | 36 | 31 | 26 | 51 | 31 | 39 | 32 |
| diameter TV | 26 | 29 | 38 | 32 | 35 | 30 | 32 | 35 | 26 | 36 | 28 | 35 | 45 | 39 | 39 | 33 |
| diameter AV | 23 | 22 | 26 | 24 | 28 | 23 | 26 | 22 | 21 | 36 | 24 | 26 | 28 | 24 | 25 | 25 |
| diameter PV | 22 | 24 | 28 | 21 | 22 | 23 | 29 | 24 | 17 | 36 | 26 | 23 | 30 | 22 | 25 | 25 |
| center MV to AV | 36 | 27 | 40 | 34 | 27 | 25 | 36 | 29 | 30 | 34 | 33 | 23 | 25 | 35 | 41 | 32 |
| center AV to PV | 36 | 33 | 40 | 37 | 31 | 39 | 37 | 32 | 27 | 31 | 31 | 31 | 40 | 44 | 33 | 35 |
| center PV to TV | 58 | 51 | 65 | 57 | 68 | 56 | 62 | 57 | 39 | 60 | 53 | 53 | 68 | 65 | 57 | 58 |
| center TV to MV | 59 | 62 | 54 | 57 | 50 | 67 | 55 | 53 | 39 | 56 | 46 | 49 | 62 | 65 | 50 | 55 |
| angle beta | 153 | 169 | 148 | 128 | 118 | 154 | 176 | 123 | 129 | 159 | 159 | 147 | 168 | 137 | 155 | 148 |
| apex-MV | 66 | 80 | 87 | 55 | 62 | 77 | 111 | 88 | 64 | 61 | 90 | 84 | 81 | 102 | 102 | 81 |
| apex-TV | 96 | 107 | 122 | 106 | 92 | 106 | 85 | 105 | 78 | 94 | 79 | 100 | 123 | 101 | 115 | 100 |
| apex-AV | 79 | 88 | 102 | 76 | 82 | 86 | 102 | 102 | 79 | 78 | 99 | 85 | 84 | 111 | 122 | 92 |
| apex-PV | 84 | 100 | 110 | 91 | 88 | 99 | 97 | 110 | 86 | 93 | 95 | 71 | 100 | 104 | 115 | 96 |
| longitudinal axis | 109 | 130 | 121 | 83 | 86 | 104 | 129 | 111 | 78 | 89 | 109 | 117 | 105 | 118 | 103 | 106 |
| TV to posterior surface | 36 | 31 | 39 | 31 | 49 | 28 |  | 46 | 41 | 45 | 48 | 17 | 37 | 35 | 33 | 37 |
| MV to posterior surface | 32 | 38 | 48 | 39 | 36 | 34 | 34 | 38 | 47 | 34 | 29 | 27 | 50 | 33 | 35 | 37 |
| sternal length | N/A | 125 | 152 | N/A | 141 | N/A | 125 | N/A | 120 | N/A | 100 | 126 | 155 | 129 | N/A | 130 |

Appendix A part 2: male patients

| **sex / patient_ID** | **male1** | **male2** | **male3** | **male4** | **male5** | **male6** | **male7** | **male8** | **male9** | **male10** | **male11** | **male12** | **Avg. Male** | **Avg. Total** |
| --- | --- | --- | --- | --- | --- | --- | --- | --- | --- | --- | --- | --- | --- | --- |
| **group** | **av** | **av** | **av** | **av** | **av** | **no hx** | **no hx** | **no hx** | **no hx** | **mcs** | **mcs** | **mcs** | **n=12** | **n=27** |
| **age** | **75** | **87** | **78** | **86** | **70** | **NA** | **36** | **49** | **83** | **45** | **65** | **66** | **67** | **76** |
| **scan resolution** | **0,45** | **0,59** | **0,46** | **0,45** | **0,59** | **0,47** | **0,43** | **0,43** | **0,70** | **0,43** | **0,38** | **0,51** | **0,49** | **0,47** |
| **slice Increment** | **0,3** | **0,3** | **0,3** | **0,3** | **0,3** | **0,5** | **0,5** | **0,5** | **1,25** | **2,5** | **0,5** | **0,5** | **0,65** | **0,57** |
| aortic valve-diaphragm | 69 | 71 | 62 | 81 | 141 | 95 | 83 | 91 | 92 | 99 | 79 | 69 | 85 | 77 |
| remnant aorta-diaphragm | 134 | 121 | 112 | 131 | 95 | 138 | 112 | 118 | 128 | 154 | 138 | 149 | 131 | 126 |
| right to left edge pericard | 100 | 123 | 133 | 123 | 143 | 98 | 122 | 120 | 134 | 160 | 114 | 170 | 128 | 129 |
| ventral-dorsal | 157 | 145 | 170 | 157 | 130 | 146 | 77 | 117 | 170 | 156 | 163 | 190 | 148 | 138 |
| TH10 – sternum | 184 | 165 | 198 | 159 | 137 | 155 | 90 | 125 | 156 | 155 | 174 | 161 | 155 | 141 |
| diameter MV | 25 | 26 | 35 | 28 | 33 | 21 | 24 | 33 | 34 | 35 | 31 | 36 | 29 | 30 |
| diameter TV | 35 | 27 | 37 | 40 | 31 | 24 | 34 | 42 | 44 | 35 | 48 | 54 | 36 | 35 |
| diameter AV | 31 | 30 | 28 | 23 | 30 | 24 | 27 | 25 | 29 | 23 | 25 | 23 | 26 | 25 |
| diameter PV | 30 | 25 | 28 | 23 | 27 | 19 | 17 | 23 | 29 | 29 | 21 | 23 | 23 | 24 |
| center MV to AV | 32 | 23 | 33 | 29 | 29 | 35 | 38 | 37 | 47 | 30 | 47 | 27 | 33 | 32 |
| center AV to PV | 35 | 32 | 44 | 32 | 32 | 26 | 41 | 28 | 32 | 43 | 35 | 52 | 36 | 35 |
| center PV to TV | 56 | 55 | 71 | 58 | 62 | 59 | 72 | 68 | 57 | 86 | 69 | 72 | 64 | 61 |
| center TV to MV | 47 | 37 | 71 | 46 | 54 | 40 | 48 | 58 | 67 | 64 | 41 | 62 | 51 | 53 |
| angle beta | 141 | 119 | 151 | 157 | 171 | 144 | 139 | 142 | 151 | 150 | 153 | 177 | 150 | 149 |
| apex-MV | 99 | 85 | 82 | 98 | 104 | 94 | 77 | 87 | 85 | 113 | 103 | 111 | 96 | 88 |
| apex-TV | 102 | 99 | 125 | 104 | 112 | 89 | 99 | 104 | 99 | 111 | 104 | 130 | 105 | 103 |
| apex-AV | 107 | 94 | 105 | 109 | 109 | 104 | 106 | 104 | 104 | 113 | 119 | 110 | 106 | 99 |
| apex-PV | 93 | 92 | 119 | 105 | 114 | 100 | 106 | 95 | 101 | 127 | 106 | 122 | 106 | 101 |
| longitudinal axis | 108 | 88 | 121 | 110 | 111 | 91 | 99 | 97 | 110 | 127 | 103 | 131 | 108 | 106 |
| TV to posterior surface | 74 | 48 | 47 | 75 | 49 | 31 | 45 | 22 | 36 | 43 | 28 | 44 | 46 | 41 |
| MV to posterior surface | 37 | 37 | 45 | 32 | 38 | 45 | 53 | 30 | 25 | 38 | 33 | 48 | 37 | 37 |
| sternal length | N/A | 129 | 144 | 167 | 194 | 168 | 132 | N/A | N/A | N/A | 181 | 154 | 159 | 144 |
