# Supplementary material for: Image based evaluation of mediastinal constraints for the development of a pulsatile total artificial heart
Source: Biomed Eng Online. 2013 Aug 14;12:81. doi: 10.1186/1475-925X-12-81 (PMC3751639; doi:10.1186/1475-925X-12-81)
Supplement: Additional file 2: Appendix B — Summary of data found in literature. [file 1475-925X-12-81-S2.docx]

Appendix B: Summary of data found in literature

| **paper** | **Shah** | **Shiono** | **Chatel** | **Komodo92** | **Komoda92b** | **Komoda95** | **Mussivand** | **Uyama** | **Pantalos** | **Fujimoto** | **Fukamachi** | **Avg total** | **Std dev** |
| --- | --- | --- | --- | --- | --- | --- | --- | --- | --- | --- | --- | --- | --- |
| **patients (n)** | **12** | **26** | **15** | **3** | **6** | **5** | **19** | **32** | **13** | **9** | **33(fit group)** | **173** | **in %** |
| aortic valve-diaphragm | x | x | x | x | 78 | x | 86 | x | 121 | 97 | x | 108 | 22 |
| remnant aorta-diaphragm | 133 | 130 | x | x | x | x | x | x | x | x | x | 132 | 1 |
| right to left edge pericard | 154 | 149 | x | x | 76 | x | x | x | 203 | 181 | x | 153 | 31 |
| ventral-dorsal | 129 | 131 | 140 | 94 | 90 | 81 | 106 | x | 135 | 123 | 150 | 118 | 20 |
| diameter MV | 30 | 29 | 31 | 23 | x | 24 | x | x | x | x | 27 | 27 | 12 |
| diameter TV | 35 | 35 | x | x | x | x | x | 44*40 | x | x | 37 | 35 | 4 |
| diameter AV | 36 | 36 | x | x | x | x | x | 59*41 | x | x | 39 | 37 | 4 |
| diameter PV | 29 | 28 | 31 | 27 | x | 28 | x | x | x | x | x | 28 | 5 |
| center MV to AV | 42 | 40 | 38 | 49 | 24 | 47 | x | x | x | x | x | 40 | 22 |
| center AV to PV | 30 | 31 | 29 | 34 | 30 | 35 | x | x | x | x | 35 | 32 | 8 |
| center PV to TV | 59 | 59 | 68 | 75 | 59 | 79 | x | x | x | x | 56 | 65 | 14 |
| center TV to MV | 52 | 53 | 65 | 47 | 48 | 47 | x | x | x | x | x | 52 | 14 |
| angle beta | 158 | 158 | 160 | 157 | 164 | 167 | x | 160 | x | x | x | 160 | 2 |
| apex-MV | x | x | 111 | 103 | 38 | 98 | x | x | x | x | 56 | 81 | 40 |
| apex-TV | x | x | 110 | 104 | 29 | 101 | x | x | 121 | 121 | 54 | 91 | 39 |
| apex-AV | x | x | 120 | 128 | 54 | 125 | x | x | x | x | 85 | 102 | 31 |
| apex-PV | x | x | 116 | 140 | 78 | 138 | x | x | x | x | 94 | 113 | 24 |
| longitudinal axis | 123 | 121 | 105 | 101 | 97 | 97 | x | x | x | x | 110 | 108 | 10 |
| center TV to posterior surface | 48 | 49 | x | x | x | x | x | x | x | x | x | 49 | 0 |
| center MV to posterior surface | 42 | 42 | x | x | x | x | x | x | x | x | x | 42 | 0 |
| sternal length | x | x | x | x | x | x | 189 | x | x | x | 199 | 194 | 4 |

Data from:

Shah AS, Shiono M, Jikuya T, Takatani S, Sekela ME, Noon GP, et al. Intraoperative determination of mediastinal constraints for a total artificial heart. *ASAIO Trans*. 1991;37(2):76–79.

Shiono M, Shah AS, Sasaki T, Takatani S, Sekela ME, Noon GP, et al. Anatomic fit study for development of a one piece total artificial heart. *ASAIO Trans*. 1991;37(3):M254–M255.

Chatel D, Martin-Bouyer Y, Vicaut E, Bouchoucha H, Achard F, Sablayrolles JL, et al. Criteria for anatomical compatibility of the total artificial heart: computerized three-dimensional modeling of the cardiovascular anatomy. *Artif Organs*. 1993 Dec;17(12):1022–1035.

Komoda T, Uyama C, Maeta H, Ozaki K. Study of anatomic constraints using three dimensionally reconstructed images for total artificial heart implantation. *ASAIO J*. 1992;38(3):M564–M569.

Komoda T, Uyama C, Maeta H, Sanou K. Three-dimensional imaging of mitral and tricuspid annuli for total artificial heart implantation. *Artif Organs*. 1992 Oct;16(5):496–501.

Komoda T, Uyama C, Maeta H, Ozaki K. Study of cardiac structures in relation to the pericardial cavity for total artificial heart implantation. *Artif Organs*. 1995 Feb;19(2):178–184.

Mussivand T, Masters RG, Hendry PJ, Rajagopalan K, Walley VM, Nahon D, et al. Critical anatomic dimensions for intrathoracic circulatory assist devices*. Artif Organs*. 1992 Jun;16(3):281–285.

Uyama C, Akutsu T. Three-dimensional interface geometry of the human heart with the artificial heart. *ASAIO Trans*. 1991;37(4):608–614.

Pantalos GM, Richenbacher WE, Karwande SV, Gay WA. Determination of critical pericardial dimensions in patients with dilated cardiomyopathy. *ASAIO Trans*. 1991;37(3):M252–M253.

Fujimoto LK, Jacobs G, Przybysz J, Collins S, Meaney T, Smith WA, et al. Human thoracic anatomy based on computed tomography for development of a totally implantable left ventricular assist system. *Artif Organs*. 1984 Nov;8(4):436–444.

Fukamachi K, McCarthy PM, Vargo R, Massiello AL, Chen JF, Byerman BP, et al. Anatomic fitting studies of a total artificial heart in heart transplant recipients. Critical dimensions and prediction of fit*. ASAIO J*. 1996;42(5):M337–M342.
